# The Code of Ethics

**Published:** 1867-09

**Authors:** 


### Editorial.

### THE CODE OF ETHICS.

All our readers are aware of the fact that the American Dental Association, at its meeting in Boston last year, adopted a code of ethics, a draft of which was prepared by a committee of three members appointed by the Association. The report of that committee was discussed, amended and adopted, and is now to all intents and purposes the code of ethics of the American Dental Association.

Within a year after its adoption by this body many local societies also adopted it, and several have had occasion to avail themselves of its instrumentality for their purification, and in every case with the most happy results. This code consists simply of plain and obvious rules and regulations of professional conduct and demeanor.

We think it is of such character that no truly professional man could object to any of its provisions. The empiric and quack do object, and strongly too, as bad men will always object to wholesome laws.

This code now stands, and will till it is revoked, as a part of the law of this national association, and is binding upon all its members as certainly as any of its other provisions; and yet, though strange it may seem, the same body, at its recent annual meeting in Cincinnati, refused to require or even recommend, local societies that are to be represented in this body, to adopt its code of ethics.

Is the American Dental Association ashamed of a part of its own good and wholesome laws and regulations? Or is it desirable to have some members in that Association who will not conform to the code of ethics? But every one who becomes a member, becomes subject to that code, as certainly and as definitely as though he had been present, and voted for it at the time of its adoption, just as clearly as he is subject to its constitution.

The formation and adoption of this code does not in one iota, nor was it intended to restrict in any sense, any just and honorable man in the profession. Good laws are made, in one sense, for transgressors, and they only contemn and despise them.

One object aimed at in the adoption of this code, was that the profession might have uniformity and harmony in this respect. The greater uniformity there is in the rules and regulations of any association or body of men, the greater will be their strength. Division and diversity is the embodiment of weakness.

At the recent meeting, one of the arguments presented against the recommendation or requirement proposed was, "that the American Dental Association has no power to determine who shall be its members, nor to define their character," and this too, notwithstanding the constitution says, "the members of this Association shall be exclusively practitioners of Dentistry," and the by-laws in the third section say, "any act of special immorality or unprofessional conduct, committed by a member of this Association shall be referred to the Committee of Arrangements, whose duty it shall be to thoroughly examine into the case and report at the next meeting, if the charges be sustained. Whereupon, by vote, the offending member may be reprimanded or expelled, a two-thirds vote being required for expulsion, a plurality being sufficient for reprimand."

Thus the falsity of that argument appears from the very letter of the law itself. And again, reason and fact both teach us, and every one knows, that there is no association or associated body of men extant, that does not reserve to itself the right to determine the character of its members. This is true all the way from the highest to the lowest, and from the best to the worst.

Again, it was said "the matter of determining who should be members, or their character, should be left entirely in the hands of local societies."

This argument is equally fallacious with the other, both in theory and fact. It is false in theory, for the very means of confining all such determinations to local societies was refused, by not requiring all societies having a representation in this body, to adopt the code of ethics. The responsibility of excluding all unfit members from this body, would be thrown upon local societies, had the Association adopted the amendment under consideration. Again, the argument was falsified by the Association within a few moments of its advancement; for about that time a committee of five was appointed to examine and report upon the fitness or unfitness of one who had been delegated to become a member, and they did examine, and thoroughly too, and made a most scathing report, refusing the delegate admission, and involving not only him, but all those associated with him, as unfit to become members of this Association; and this report was unanimously adopted by this Association, after having voted against the proposed amendment, influenced no doubt by the arguments quoted above, and worse ones. One prominent member said he had never heard of a code of ethics having been adopted, till within a very short time, and had never seen it, therefore he should vote against it. Query? Was he prepared to vote intelligently against it?

A very respectable number of our local societies, we think a majority, have adopted the code of ethics, some have not; two, we believe, have rejected it. We cannot but wish they occupied a different position, in reference to which we may have something to say in future.

